# Journal of Automatic Chemistry: Editorial Board

**DOI:** 10.1155/S1463924691000408

**Published:** 1991


Journal of Automatic Chemistry, Vol. 13, No. 6 (November–December 1991), pp. 241–242

### Journal of Automatic Chemistry: Editorial Board

Continuing *JAC*'s series of introductions to its editorial team, we are pleased to publish short biographies of four of our clinical editors:

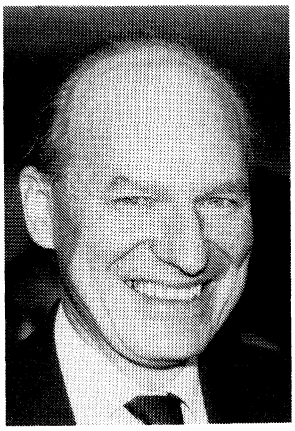

Christian Collombel is Professor of Biomedical Engineering at the University Claude Bernard (Lyons) and is also director of the clinical biochemistry laboratory at the emergency hospital in Lyons (Hôpital Edouard Herriot).

He obtained his PhD in biochemistry in 1962 and in pharmaceutical sciences in 1968, both from the University of Lyons. He has been active in the Commission of Instrumentation of the French Society of Biological Chemistry (President, 1973–1983) and in the Expert Panel of Instrumentation of IFCC (1975–1981). Currently he is responsible for developing bilateral relationships between research and industry in the field of biomedical engineering, in the Rhone Alpes area. He is also responsible for doctoral education in the discipline of biomedical engineering in the universities of the Rhone Alpes.

He is a member of the European Commission of Normalisation for Bioreagents and Analyser instruments, and a member of the commission of homologation for the French Ministry of Health.

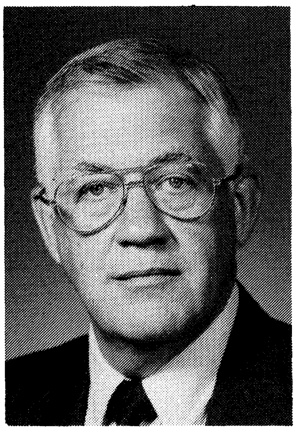

Carl A. Burtis is Chief of Clinical Chemistry in the Chemical Technology and Health Divisions at the Oak Ridge National Laboratory in Oak Ridge, Tennessee, USA. He received his BS degree from Colorado State in 1959, and MS and PhD degrees from Purdue University in 1965 and 1976, respectively. He has been very active in many professional organizations, including AACC, NCCLS, IFCC and IMPAC. In 1989, he served as President of the AACC and in 1990 as Chairman of the Organizing Committee for the 14th International Congress of Clinical Chemistry.

His research interests include: HPLC, centrifugal analysis, standardization of clinical methods, and clinical analysis in general. In addition to his association with the *Journal of Automatic Chemistry*, he also serves on the editorial boards of *Clinical Chemistry* and the *Giornale Italiaro di Patologia Clinica.*

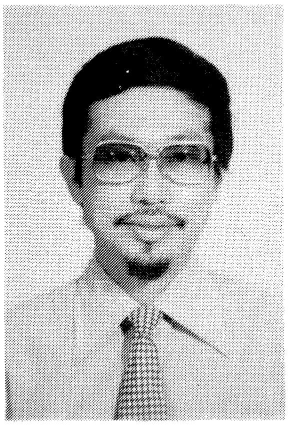

It-Koon Tan is qualified as a PhD and holds fellowships of the Royal Society of Chemistry and the Royal College of Pathologists, UK, and the Singapore National Institute of Chemistry, along with the Mastership in Clinical Biochemistry, UK. He is head of the division of biochemistry in the Department of Pathology which provides laboratory services to the health services of Singapore. Previously he has been a part-time lecturer in clinical biochemistry at the National University in Singapore and a board member of the Singapore Science Council. He has just completed a three-year term (1988–90) as a member of the executive board of the IFCC. He is a member of the executive committee (and immediate past president) of the congress committee of the Asian and Pacific Federation of Clinical Biochemistry (APFCB).

He has a wide general interest in clinical chemistry, having published more than 90 papers, and is currently on the editorial boards of the *APFCB Newsletter*, the *Asian Journal of Clinical Sciences*, *Clinica Chimica Acta* and *Advances in Clinical Chemistry*. He has been extensively involved in the development of clinical biochemistry in the Asian and Oceanic countries over the last 20 years.

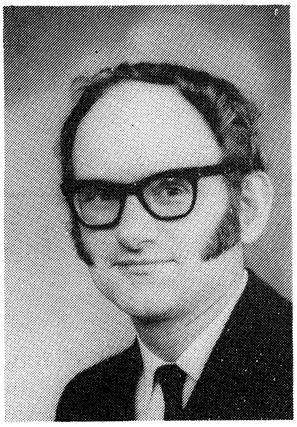

Barry Roberts is a fellow of the Royal Society of Chemistry, UK, obtained a PhD from the University of Edinburgh in 1973 and the Mastership in Clinical Biochemistry, UK, in 1969.

He is currently deputy head at the Department of Pathological Biochemistry at the Western Infirmary and Gartnavel General Hospitals in Glasgow and has been employed at several National Health Service teaching hospitals over the last 20 years. He has held full-time and honorary lectureships at the Universities of Edinburgh, Hull and Glasgow, respectively. He was a past secretary of the instrumentation committee of the Association of Clinical Biochemists, UK, and is a member of the Equipment Evaluation Panel for the Scottish Health Services. His topical interests are in robotics and expert systems.

